# A Meta-Surface Antenna Array Decoupling (MAAD) Method for Mutual Coupling Reduction in a MIMO Antenna System

**DOI:** 10.1038/s41598-018-21619-z

**Published:** 2018-02-16

**Authors:** Ziyang Wang, Luyu Zhao, Yuanming Cai, Shufeng Zheng, Yingzeng Yin

**Affiliations:** Xidian University, National Key Laboratory of Antennas and Microwave Technology, Xi’an, 710071 People’s Republic of China

## Abstract

In this paper, a method to reduce the inevitable mutual coupling between antennas in an extremely closely spaced two-element MIMO antenna array is proposed. A suspended meta-surface composed periodic square split ring resonators (SRRs) is placed above the antenna array for decoupling. The meta-surface is equivalent to a negative permeability medium, along which wave propagation is rejected. By properly designing the rejection frequency band of the SRR unit, the mutual coupling between the antenna elements in the MIMO antenna system can be significantly reduced. Two prototypes of microstrip antenna arrays at 5.8 GHz band with and without the metasurface have been fabricated and measured. The matching bandwidths of antennas with reflection coefficient smaller than −15 dB for the arrays without and with the metasurface are 360 MHz and 900 MHz respectively. Using the meta-surface, the isolation between elements is increased from around 8 dB to more than 27 dB within the band of interest. Meanwhile, the total efficiency and peak gain of each element, the envelope correlation coefficient (ECC) between the two elements are also improved by considerable amounts. All the results demonstrate that the proposed method is very efficient for enhancing the performance of MIMO antenna arrays.

## Introduction

The surge in video traffic as well as the number of mobile terminal users have boosted the development in wireless communication technologies and systems. As the whole world is moving toward the era of 5-th generation mobile communication, the demand for even higher spectrum and energy efficiency will be everlasting^1^.

In this circumstance, the MIMO/Massive MIMO technology has become an indispensable part of the future wireless communication systems. MIMO technology has the potential to significantly enhance spectrum efficiency by utilizing multiple yet independent data streams existing between the transmitter and the receiver where multiple antennas are installed^2^. While Massive MIMO takes a step further, it uses antenna array with a few hundred elements simultaneously to serve dozens of mobile terminal in limited spectrum and time^1^. In practical applications where space is always limited, antenna arrays, either at the base station end or in a mobile terminal, must be compact in size. Therefore, the inter-element distance is only a fraction of wavelengths, causing inevitable mutual coupling. Mutual coupling will degrade the array performance in the following aspects:

1. Side-lobes might occur and it will affect the beam-scanning capability of the array^3,4^.
2. The SNR of the array will be deteriorated as unwanted coupling between elements will cause data streams received by other antenna elements rather than radiating into free space^2^.
3. The envelop correlations between elements become high thus data throughput is significantly reduced^5,6^.
4. Gain and total efficiency of the array will drop since effective radiated power are coupled elsewhere and dissipated on the loads of the antenna elements^2^.

Consequently, the mutual coupling reduction technique, or termed as the antenna decoupling technique has drawn great attention from both the academia as well as the industry. The existing and developing decoupling solutions include: neutralization lines^7,8^; decoupling with parasitic scatters^9,10^; eigen-mode decomposition techniques^11–14^ and passive decoupling networks^15–19^. However, only a few of them can be readily extended to Massive MIMO antenna arrays with dozens of elements. Metamaterial based or inspired solutions^20–25^ are also becoming popular, but most of them are very effective for arrays with limited number of antennas where inter-element spacing is moderate (no less than 0.1 ${{\rm{\lambda }}}_{0}$).

In the paper, a metasurface suspended over an antenna array is proposed for antenna decoupling purpose. The idea of covering a metasurface over an antenna for performance enhancement is not new, but most of them are not used for array antenna decoupling, they are used

- To form a Fabry-Perot resonator type antenna for gain enhancement^26–28^.
- For low-profile antenna (array) design^29^.
- To reduce the Radar Cross Section (RCS) of the antenna^30^.
- For beamforming or beam scanning applications^31,32^.

To the best of the authors’ knowledge, it is for the first time that a metasurface covering with negative permeability characteristic is used for antenna decoupling. The proposed MAAD method for compact antenna arrays utilizing meta-surface cover possess the following remarkable features:

1. Both the decoupling and matching bandwidths of the array with the metasurface are very broad without any extra matching measures;
2. It can be applied to compact antenna arrays where inter-element spacing is extremely small (smaller than 0.02 ${{\rm{\lambda }}}_{0}$);
3. Because of the periodic nature of metasurfaces, it has great potential to be applied to Massive MIMO antenna arrays where a few hundreds of antennas are excited simultaneously;
4. It is also able to enhance gain and efficiency of the antenna array thanks to the focusing capability of the metasurface.

## Design Method

### Antenna Array Decoupling Mechanism in a Negative Permeability Medium

**Figure 1:**
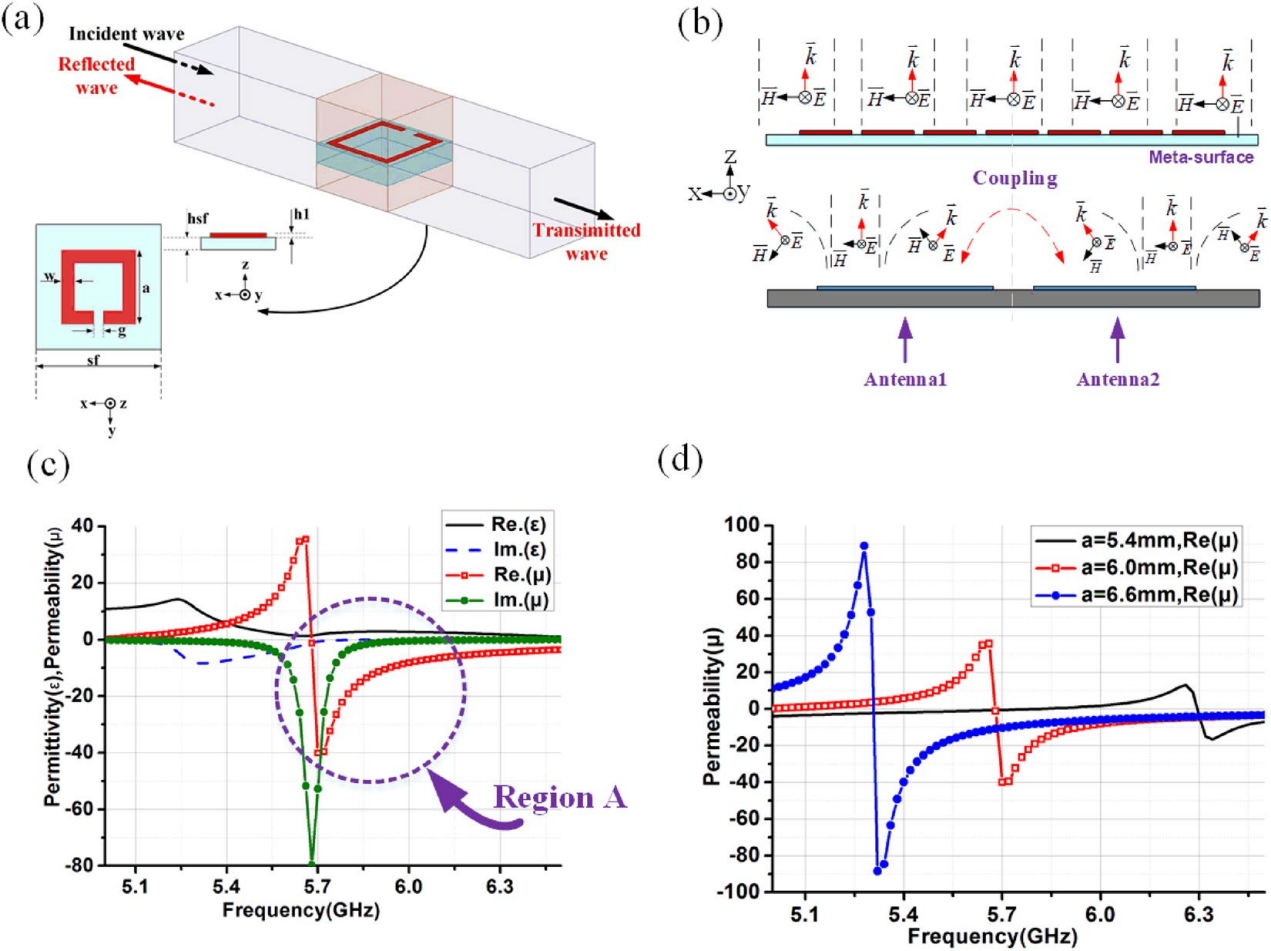
(**a**) The electromagnetic model to extract design parameters; (**b**) Radiated field distribution of two coupled antenna elements in an antenna array with the metasurface. (**c**) The permittivity and permeability of SRR unit; and (**d**) Simulated permeability of SRR unit with respect to different *a* values.

As shown in Fig. 1 (a) and (b), a suspended meta-surface with SRRs as its unit cell over a two-element antenna array is used to illustrate the working mechanism of the MAAD method. An isotropic homogeneous slab model (Fig. 1a) is used to calculate the effective permittivity, the effective permeability of the metasurface unit using the extraction methods well-known^33–36^. The resonant frequency and the negative permeability frequency region of the SRRs are determined by the physical dimension of the ring and the size of the gap as shown in Table 1. The equivalent values of permittivity and permeability for the proposed SRRs unit are shown in Fig. 1(c). As shown in Fig. 1(c), in 5.8 GHz frequency band, the real part of permittivity is positive, while the real part of permeability is negative. It is obvious that tuning the parameter *a*, the negative permeability frequency region of the SRRs can easily be manipulated as shown in Fig. 1(d).

**Table 1:** Design Parameters of the SRR unit (Unit: mm).

Parameters	Values	Parameters	Values
*a*	6.0	*w*	0.5
*g*	1.2	*sf*	7
*h*_1_	0.035	*h*_2_	1.5

To reveal the mutual coupling reduction mechanism using the MAAD method, let’s consider a two-element MIMO antenna array shown in Fig. 1(b). When antenna 1 is excited, electromagnetic waves propagate in the way shown in Fig. 1(b), stray coupling component ${A}_{0}{e}^{jkx}$ which travels along the minus X-direction will cause induced current on antenna 2 thereby create mutual coupling between the two antennas.

A piece of suspended metasurface over the antenna array is able to create a region with negative permeability yet positive permittivity (${\mu }_{r} < 0,\,{{\epsilon }}_{r} > 0$), as shown in Fig. 1(c). In this region, the wavenumber can be expressed as:

1 $$k={k}_{0}\cdot \sqrt{-|{\mu }_{r}|\cdot |{{\epsilon }}_{r}|}=j{k}_{0}\cdot \sqrt{|{\mu }_{r}|\cdot |{{\epsilon }}_{r}|}$$

which is purely imaginary. (Only positive solution of equation (1) is valid).

In this case, the corresponding x-component of the electric field travelling along the −x direction,${A}_{0}{{\rm{e}}}^{{\rm{jkx}}}$ can be further expressed as:

2 $${A}_{0}{e}^{jkx}\cdot {e}^{j\omega t}={A}_{0}{e}^{j(j{k}_{0}\cdot \sqrt{|{\mu }_{r}|\cdot |{{\epsilon }}_{r}|})x}\cdot {e}^{j\omega t}={A}_{0}{e}^{-{k}_{0}\sqrt{|{\mu }_{r}|\cdot |{{\epsilon }}_{r}|}x}\cdot {e}^{j\omega t}$$

Equation (2) shows that electromagnetic wave travelling along minus X-direction of the metasurface is evanescent. In this manner, the wave creating mutual coupling between the two antennas is rejected. When the wave radiated by antennas propagate along Z-direction, while the magnetic field component is in the X-direction, radiation is assured by the anisotropic nature of the metasurface.

#### Design of two-element Microstrip Antenna Array with a Metasurface

**Figure 2:**
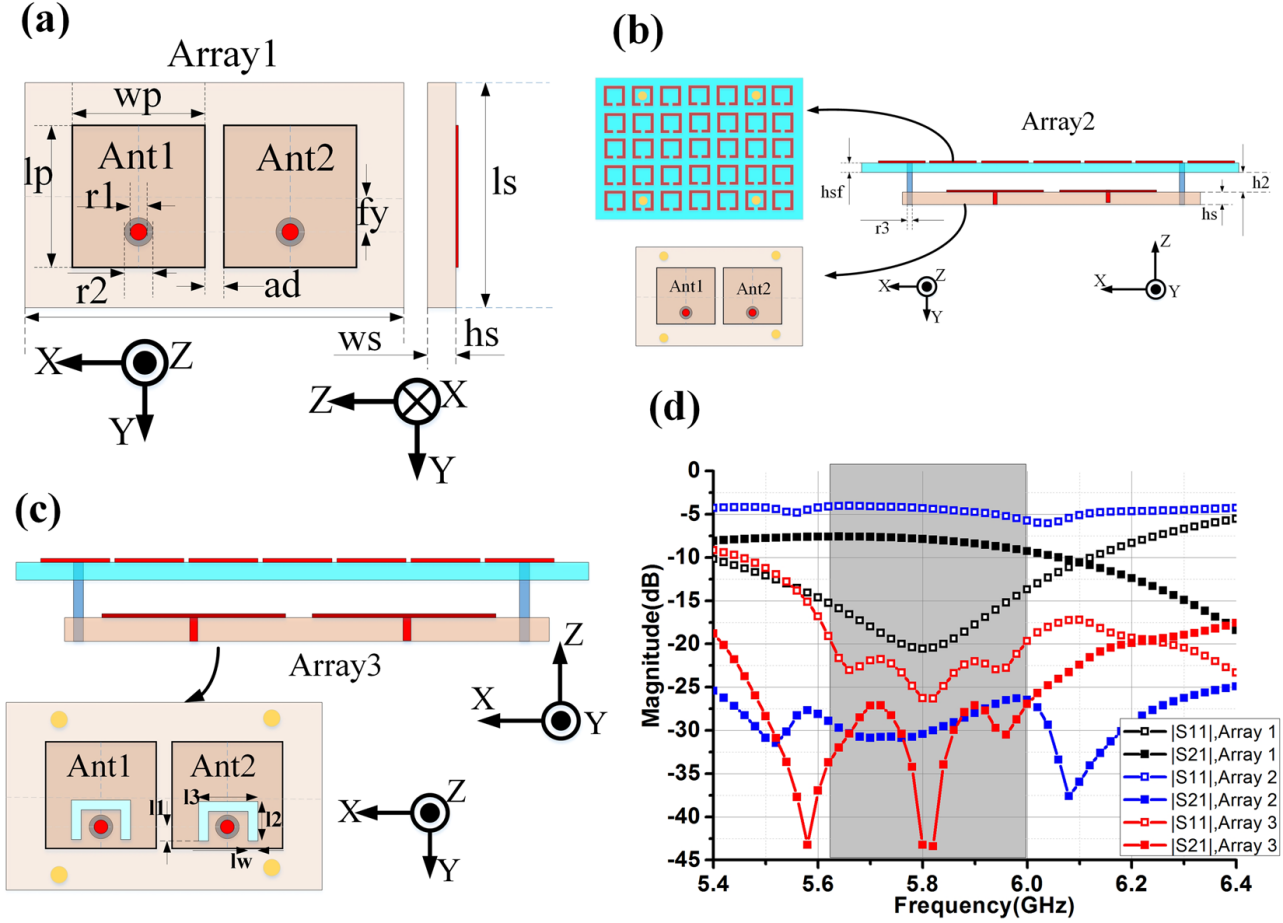
(**a**) Top view (left) and side view (right) of the two-element patch Array1; (**b**) Top view (left) and side view (right) of the two-element patch Array2 with a metasurface; (**c**) Top view (left) and side view (right) of the improved two-element patch Array3 with a metasurface; (**d**) Simulated S-parameters of the Array1, Array2 and Array3.

To further demonstrate the effectiveness of the MAAD method for mutual coupling suppression, a two-element microstrip antenna array (Array1) as shown in Fig. 2(a) is used in this paper for illustration purpose. The physical dimensions of the array are listed in Table 2. The edge-to-edge distance between the elements *ad* = 1 mm, which is approximately 0.02 ${{\rm{\lambda }}}_{0}$ at 5.8 GHz. The patches are printed on a FR4 substrate with the dielectric constant of 4.4 and the loss tangent of 0.02. The simulated S-parameters of the Array1 are plotted in Fig. 2(d). It is obvious from Fig. 2(d) that although the antenna elements are well matched at the 5.8 GHz band, but the isolation between the two antenna elements is no more than 8 dB, which is very poor. Effective decoupling measures must be taken.

**Table 2:** Design Parameters of the SRR unit (Unit: mm).

Parameters	Values	Parameters	Values	Parameters	Values
*ws*	40	*h2*	4.8	*hsf*	1.5
*ls*	26	*r1*	0.65	*lw*	0.6
*hs*	3	*r2*	1.5	*l1*	3.0
*wp*	12.4	*a*	6	*l2*	4.2
*lp*	12.5	*w*	0.5	*l3*	4
*ad*	1	*g*	1.2	—	—
*fy*	3.3	*sf*	7	—	—

A metasurface consists of periodic of SRRs is introduced to improve the inter-element isolation of the array in Fig. 2(b). The SRRs are printed on a substrate with the relative dielectric constant of 2.65 and loss tangent of 0.001. Four dielectric posts are introduced to provide mechanical support for the metasurface and the antenna array (Array2). The dimensions in Fig. 2(b) are also shown in Table 2.

Simulated S-parameters of the Array2 with the metasurface are superposed in Fig. 2(d). It can be seen from Fig. 2(d) that isolation performance is significantly enhanced by introducing the metasurface covering while matching performance is not ideal, this is understandable since the antennas are no longer matched to a free-space medium. Then two U-shape slots which is commonly used to enhance microstrip antenna matching^37^ are etched on the antenna near the feeding probe (Array3) as shown in Fig. 2(c). The matching bandwidth of Array3 with |S_11_| smaller than −15 dB is about 900 MHz, which is about 2.5 times of the Array1 without metasurface. While the isolation between the elements of Array3 is all below −27dB from 5.49 GHz to 6.0 GHz, which is increased by a large amount compared to Array1. Moreover, the profile of the Array3 with the metasurface is only increased by about 0.09 ${{\rm{\lambda }}}_{0}$, which is acceptable.

### Field Distribution

**Figure 3:**
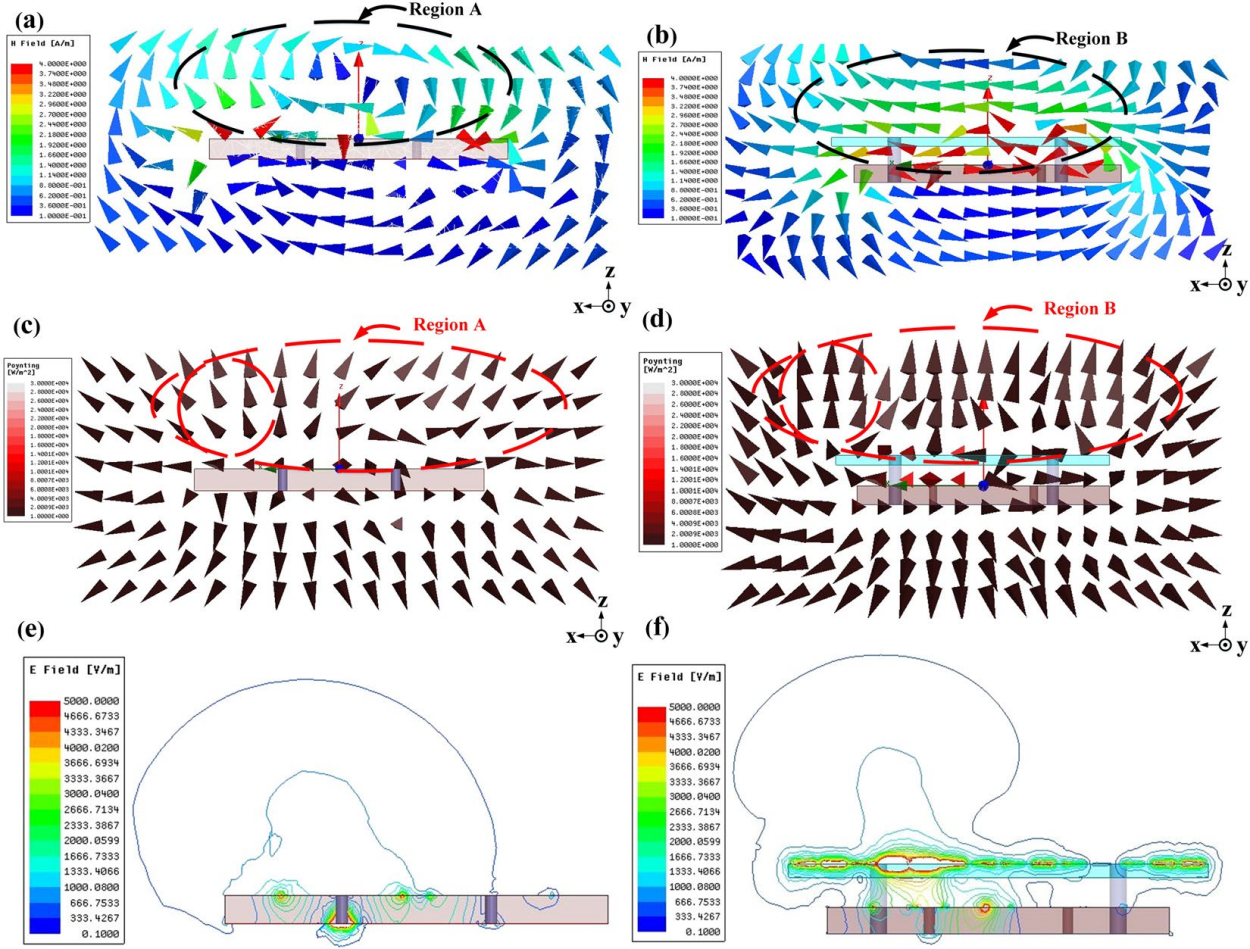
(**a**) Simulated vector magnetic field distribution on a cutting surface in the XOZ-plane for array without metasurface; (**b**) Simulated vector magnetic field distribution on a cutting surface in the XOZ-plane for array with metasurface; (**c**) Simulated Poynting vector distribution on a cutting surface in the XOZ-plane for array without metasurface; (**d**) Simulated Poynting vector distribution on a cutting surface in the XOZ-plane for array with metasurface; (**e**) Simulated electric field contours on a cutting surface in the XOZ-plane when Ant 1 is excited while Ant 2 terminated with matched load for array without metasurface; (**f**) Simulated electric field contours on a cutting surface in the XOZ-plane when Ant 1 is excited while Ant 2 terminated with matched load for array with metasurface. All of the plots are at 5.8 GHz.

To further reveal the underlying working mechanisms of the array with the metasurface, the vector magnetic field distributions on a cutting surface within XOZ-plane are investigated using ANSYS HFSS. As shown in Fig. 3(a) and (b). Without the metasurface, the vector H-field is rotating in Region A with the metasurface, the H-field in Region B is almost along the X-axis. On the same cutting plane, the Poynting vector distributions are also plotted and shown in Fig. 3(c) and (d) for the arrays without and with metasurface, respectively. The Poynting vectors for the array with metasurface are almost in parallel to the Z-axis without any lateral radiation.

The contours of the electric field for the arrays without and with metasurface are also shown in Fig. 3(e) and (f). In the simulation, Ant 1 is excited while Ant 2 is terminated with matched load. It can be concluded from Fig. 3(e) and (f) that, in the array without metasurface, stray coupling from antenna 1 will be coupled to antenna 2 and absorbed by the load. While with the help of the metasurface, the wave front concentrate toward the normal direction of the microstrip antenna.

### Parametric Studies

**Figure 4:**
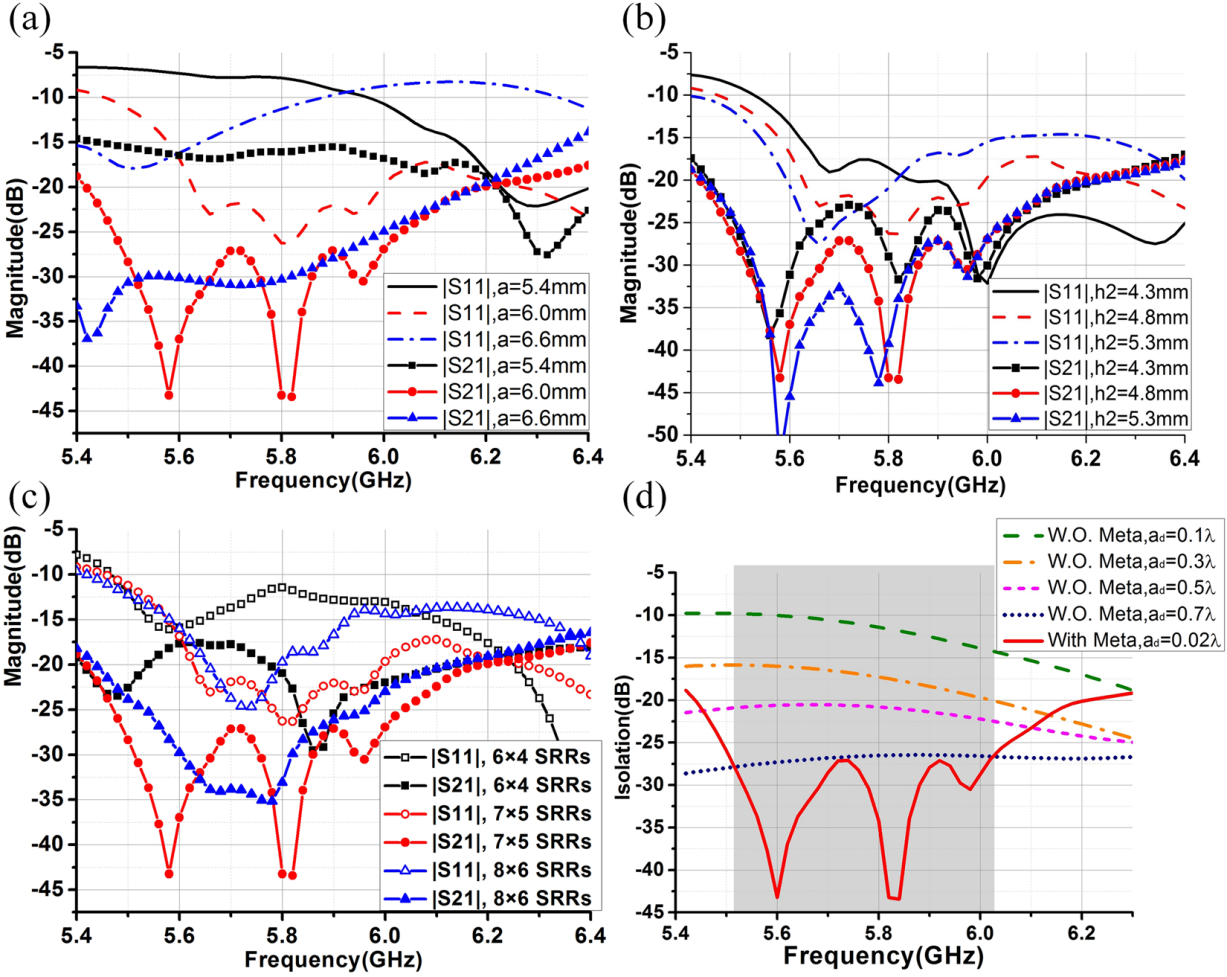
(**a**) Simulated S-parameters of the array with the metasurface with respect to different a- the length of the SRR, values; (**b**) Simulated S-parameters of the array with the metasurface with respect to different h2- the height of the metasurface, values; (**c**) Simulated S-parameters of the antenna array with the metasurface with respect to different number of SRR units; (**d**) Simulated isolations for arrays with different ad values and the isolation for the array with a metasurface.

Parametric studies are conducted to analyze the sensitivity of different design parameters. Two clusters S-parameter curves are obtained with respect to different lengths of the SRR, *a*; and different heights of the metasurface covering, *h2*. They are superposed in Fig. 4(a) and (b), respectively. It can be seen that parameter *a*, the length of the SRR is more sensitive in the design, since the length of the SRR will directly affect the negative permeability frequency band of the SRR, as shown in Fig. 1(d). Therefore, the band-rejection frequency of the metasurface will shift to lower frequencies if parameter *a* increases, and it will shift to higher frequencies if parameter *a* decreases, which is consistent with the observation in Fig. 1(d).

The isolation performance of the antenna array with the metasurface is not very height-sensitive as shown in Fig. 4(b). This is reasonable since the rejected propagation band of the metasurface remains unchanged, yet the return losses of the array vary significantly because of different parasitic effect of the metasurface with respect to different meta-surface height.

In order to explore the optimum configuration of the SRR units on the metasurface, the matching and isolation performances for the array with metasurface of 6 × 4, 8 × 6 and 7 × 5 periodic SRR units are also compared in Fig. 4(c). Form Fig. 4(c), we can conclude that the both the matching and isolation performance of the array with only 6 × 4 SRR units are not ideal. The isolation performance for the array with 8 × 6 SRR units is better than the array with 7 × 5 SRR units only within the bandwidth from about 5.65 GHz to no more than 5.8 GHz. If we look at the band from 5.6 GHz to 6 GHz, the isolation performance of the array with 7 × 5 SRR units is actually better than the array with 8 × 6 SRR units.

Furthermore, to quantify the effectiveness of the decoupling method with the metasurface, antenna array without any metasurfaces but with different edge-to-edge spacing values, *ad* are simulated. The results are superposed with the isolation for Array3 in Fig. 4(d). Within the frequency band of interest, the isolation of the array with the metasurface is equivalent to the array without the metasurface but whose inter-element spacing is about 35 times larger.

## Results

### Scattering Parameters

**Figure 5:**
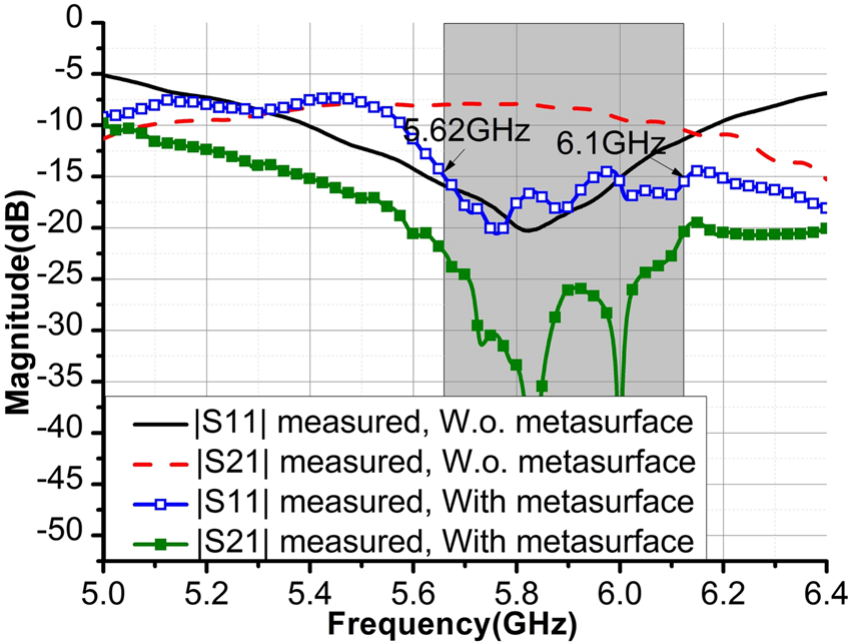
Measured S-parameters of the arrays with and without metasurface.

Both Array1 and Array3 are fabricated and measured. The scattering parameters are measured using Keysight E5080A vector network analyzer and they are superposed in Fig. 5, which is consistent with simulation results shown in Fig. 2(d). The measured highest isolation of Array3 can reach to more than 40 dB level. The measured Array3’s operating bandwidth with |S_11_| < −15 dB and |S_21_| < −25 dB is about 400 MHz.

### Radiation Parameters

**Figure 6:**
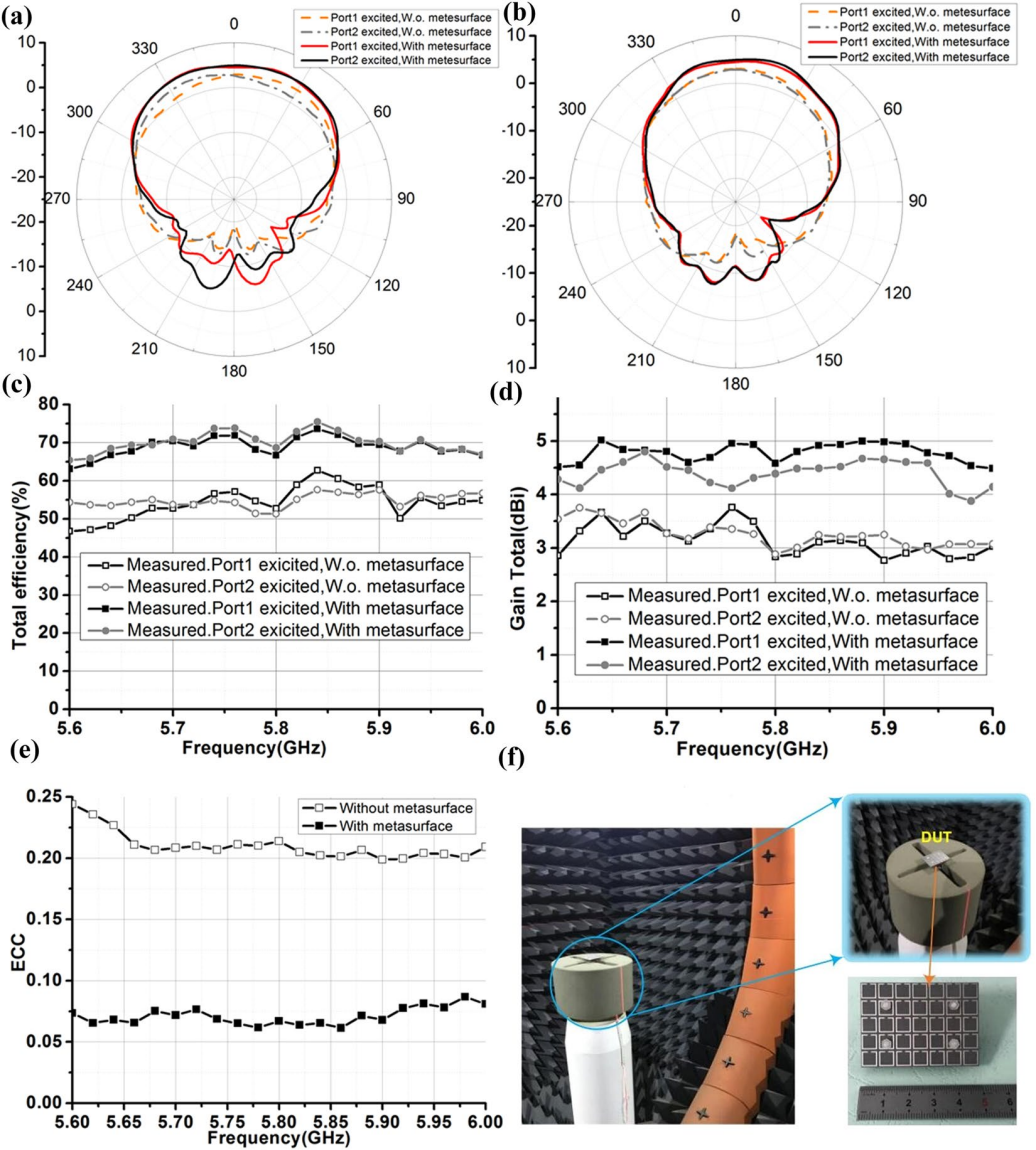
(**a**) Measured radiation patterns for the arrays at 5.8 GHz with and without metasurface in the XOZ plane; (**b**) Measured radiation patterns for the arrays at 5.8 GHz with and without metasurface in the YOZ plane; (**c**) Measured total efficiencies of the arrays with and without metasurface; (**d**) Measured peak gains of the arrays with and without metasurface on different frequencies; (**e**) Measured and calculated ECCs for the arrays with and without metasurface. (**f**) Radiation measurement setup and DUT configuration.

To further confirm the performance of the array with the metasurface, the fabricated samples of the arrays are put into the SATIMO SG-24 near field scanner (shown in Fig. 6(f)) to evaluate the radiation related characteristics, including: total efficiencies, gains, and envelope correlation coefficients. Since the mutual coupling is reduced by more than 20 dB and more powers are directed to the normal direction of the antennas, both the total efficiencies and the peak gains of the array with metasurface will be enhanced. As shown in Fig. 6(c), the total efficiency for the array with the metasurface (Array3) is 20% higher as compared to the array without any metasurfaces (Array1). The radiation patterns of the arrays are also shown in Fig. 6(a) and (b) while the peak gains with respect to different frequencies are shown in Fig. 6(d), showing around 2 dB improvement.

The envelop correlation coefficient (ECC) is an important figure of merit for any MIMO enabled antenna systems. It can be calculated from measured complex field patterns by using

3 $${\rho }_{e}=\frac{{|{\iint }_{4\pi }[\mathop{{E}_{1}}\limits^{\rightharpoonup }(\theta ,\varphi )\cdot \mathop{{E}_{2}}\limits^{\rightharpoonup }(\theta ,\varphi )]d{\rm{\Omega }}|}^{2}}{{\iint }_{4\pi }{|\mathop{{E}_{1}}\limits^{\rightharpoonup }(\theta ,\varphi )|}^{2}d{\rm{\Omega }}\cdot {\iint }_{4\pi }{|\mathop{{E}_{1}}\limits^{\rightharpoonup }(\theta ,\varphi )|}^{2}d{\rm{\Omega }}}$$

4 $$\mathop{{E}_{1}}\limits^{\rightharpoonup }(\theta ,\varphi )\cdot \mathop{{E}_{2}}\limits^{\rightharpoonup }(\theta ,\varphi )={\mathop{E}\limits^{\rightharpoonup }}_{\theta 1}(\theta ,\varphi )\cdot {\mathop{E}\limits^{\rightharpoonup }}_{\theta 2}^{\ast }(\theta ,\varphi )+{\mathop{E}\limits^{\rightharpoonup }}_{\varphi 1}(\theta ,\varphi )\cdot {\mathop{E}\limits^{\rightharpoonup }}_{\varphi 2}^{\ast }(\theta ,\varphi )$$

where $\mathop{{E}_{1}}\limits^{\rightharpoonup }(\theta ,\varphi )$ is the measured electric filed vector radiated by antenna 1 while the another antenna port is terminated by a 50Ω matched load^38^. The calculated ECCs for the arrays with and without metasurface are given in Fig. 6(e). It is clear that by introducing the metasurface, the ECC has improved from 0.25 to less than 0.08, which will definitely lead to larger channel capacity and diversity gain.

## Conclusion

A brand-new decoupling method making use of metasurface covering with negative permeability is introduced for the first time in this paper. The metasurface has the capability of reject unwanted radiation as well as reducing mutual coupling in an antenna array without affecting other performances.

The design can be conformal and low-profile, and most importantly, it can be applied to arrays with extremely small element spacing while most existing decoupling solutions cannot. Unlike other decoupling method, as isolation after decoupling becomes higher, the matching bandwidth is not reduced at all, on the contrary, it is even broader.

Thanks to the periodic nature of the metasurface with SRRs, the proposed MAAD method has great potential to be applied to antenna arrays with dozens of elements, which are commonly utilized in Massive MIMO arrays and phased arrays. Using the metasurface decoupling method proposed in this paper to decouple more than 64 element antenna array is undergoing and results will be reported on in due time.

## Methods

Numerical calculation of the decoupling antenna array was performed using Finite Element method, which is conducted by commercial software, ANSYS HFSS. The microstrip antennas are printed in a commercial FR4 printed circuit board with the relative permittivity 4.4 and loss tangent 0.02. Moreover, the substrate printed with square split ring resonators was a commercial printed circuit board (F4B) with the relative permittivity 2.65 and loss tangent 0.001. In experiments, we used the Agilent vector network analyzer to measure the transmission and reflection coefficients between antennas. The far-field radiation patterns are evaluated using a microwave near-field chamber.
